# Hernia uterine inguinale with transverse testicular ectopia and mixed germ cell tumor

**DOI:** 10.4103/0970-1591.30274

**Published:** 2007

**Authors:** Rajshekhar C. Jaka, M. Shankar

**Affiliations:** Department of Surgery, SDU Medical college and RLJ Hospital and Research Centre, Tamaka, Kolar, Karnataka - 563 101, India

**Keywords:** Hernia uterine inguinale, persistent mullerian duct syndrome, transverse testicular ectopia

## Abstract

Persistent mullerian duct syndrome is a rare disorder characterized by the presence of uterus and fallopian tube in 46XY phenotypic males and is ascribed to defects in the synthesis or action of anti-mullerian hormone. We report a rare case of hernia uterine inguinale, transverse testicular ectopia associated with mixed germ cell tumor of the testis with metastasis. Transverse testicular ectopia should be suspected preoperatively in patients who have unilateral inguinal hernia associated with contralateral nonpalpable testis. In such cases ultrasonography should be done prior to repair of hernia to evaluate the possible presence of mullerian structures and testicular malignancy, for better management.

## INTRODUCTION

Persistent mullerian duct syndrome (PMDS) is a rare autosomal recessive disorder, characterized by the persistence of mullerian duct derivatives (i.e. uterus, fallopian tubes and upper two-thirds of vagina) in otherwise normally differentiated 46 XY males. During embryogenesis, regression of mullerian structures in normal males is mediated by antimullerian hormone (AMH), also called mullerian inhibiting substance (MIS), produced by fetal sertoli cells. This internal pseudohermaphroditism is caused by deficient AMH or abnormal AMH Type II receptor. These patients often present with cryptoirchidism or an inguinal hernia. Presence of uterus in the hernial sac is known as ‘hernia uterine inguinale’.

Transverse testicular ectopia (TTE) is a rare form of testicular ectopia in which both testes are located on one inguinal side. One testis crosses midline to meet its opposite mate. As with undescended testis, these gonads are at increased risk of malignant transformation. Persistent mullerian duct syndrome exists with one-third cases of transverse testicular ectopia. We report a rare case of right side hernia uterine inguinale with TTE and advanced metastatic mixed germ cell tumor.

## CASE REPORT

A 35-year-old male patient came with a history of right-sided inguinal hernia for the past 15 years, which became irreducible and painful since six days. He complained of severe back pain since three months. He was married for 15 years and had no sexual dysfunction. He had two children. There was no history of any drug intake by his mother during the first trimester of pregnancy. There was no family history of such disorder.

On physical examination, secondary male sexual characters were found to be well developed and had a normal penis. Right inguinal swelling was 8×6cm in size, tender and irreducible. The scrotum was well developed on the right side with soft testis in it and left side was poorly developed without a palpable testis. Rest of the physical examination was unremarkable. Clinical diagnosis of right-sided irreducible inguinal hernia with left cryptoirchidism was made.

**Figure 1:**
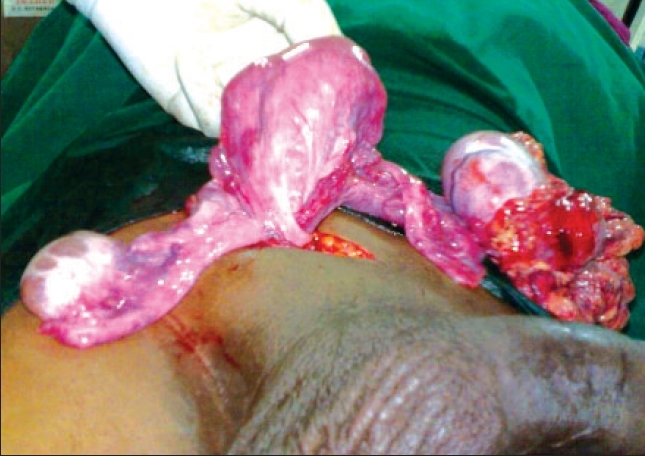
Intra operative image showing hernial sac containing uterus, fallopian tubes and germ cell tumor

Patient was operated upon under spinal anesthesia. Peroperatively, the hernial sac contained a uterus-like mass with a tubular structure on each side [Figure 1]. One side of tube was attached to a 7 × 6 × 3.5 cm white, hard, irregular mass which was attached to the omentum. On pulling the cord structures, normal-appearing testis delivered from the right side of the scrotum. The cord was separated meticulously from the rest and the uterus along with the mass removed in toto. Right testis was replaced into the scrotum and hernia repaired by polypropylene. Postoperative course was uneventful.

Histology revealed normal hypoplastic uterine tissue with attached fallopian tube. The mass was of malignant germ cell tumor with predominance of yolk sac elements. Additional components of embryonal carcinoma and teratoma were seen. Tumor was found to infiltrate spermatic cord. Few vascular emboli were also seen in the capsule. There was no ovarian tissue. Chromosomal analysis revealed 46 XY. Scan of the abdomen revealed multiple liver secondaries with multiple para-aortic and bilateral para-ileac lymph node involvement. There was thrombosis in the infrarenal aspect of the IVC and bilateral common ileac veins. Associated left side hydronephrosis was found. Right scrotal testis was normal. Chest X-ray was normal. In view of raised AFP, bHCG and LDH and nonpulmonary visceral metastasis, tumor was graded as poor risk Stage III. Even with anticoagulant therapy, DVT progressed relentlessly and he died of pulmonary embolism before the first cycle of BEP chemotherapy could be completed.

## DISCUSSION

Müllerian (paramesonephric) ducts and wolffian (mesonephric) ducts are the anlagen of the female and male reproductive tracts, respectively. In the XY fetus, the testis differentiates by the end of the seventh gestational week. Sertoli cells begin to secrete AMH, which is responsible for the regression of the Müllerian ducts. The AMH binds to a specific Type II serine-threonine kinase transmembrane receptor (AMHR-II). Human AMH gene localized near the tip of Chromosome 19, AMHR2 gene is located on 12q13. The type of persistent Mullerian duct syndrome caused by mutation in the AMH gene will be referred to as Type I, that which forms due to mutation in the AMH receptor (AMHR) will be designated as Type II.[1] In 45%, a mutation of the anti-mullerian hormone (AMH) gene was detected; in 39% mutation of the Type II receptor of AMH was detected; in 16% the cause is unknown.[2]

It is not necessary to perform testicular biopsy to detect tumor in the scrotal testis, because an impalpable tumor can be localized by ultrasonography. Orchidectomy of ectopic testis should be done, because orchidopexy offers only limited protection against future malignancy if performed after two years of age.[3] Manassero *et al* reported development of mixed germ cell tumor 18 years after bilateral orchidopexy.[4] Most are known to be infertile but our patient had two children and in such cases it is preferable to remove ectopic testis, as it is prone for malignancy. If this is necessary on both sides, there is the additional problem of lifelong testosterone substitution which requires efficient patient monitoring and good patient compliance. In cases where this cannot be achieved, compromises, such as temporarily delayed orchidectomy, may be considered.[5] Testis, vas and epididymis are closely adherent running along the uterus and fallopian tubes. This gives rise to difficulty in separating the gonads and the vas without damage. Different surgical methods have been described for safe surgery. There have been at least three documented reports of adenocarcinoma in the mullerian duct remnants. So, contrary to previous suggestions, now it is recommended to remove the persistent mullerian derivatives. The patient or his family should be completely informed of the diagnosis, the surgical options and the need for long-term follow-up. Finally, genetic counseling must be offered to the patient or his parents because of the possible chromosomal origin of the syndrome.

Many diagnostic mistakes are made which could be prevented by performing abdominopelvic ultrasonography before surgical treatment of inguinal hernia with contralateral nonpalpable testis for better management. The patient or his relatives should be educated to come early in the course of such conditions so that malignant change can be prevented by early surgery or detected in early stages and prognosis can be improved.
